# DEC205 mediates local and systemic immune responses to *Helicobacter pylori* infection in humans

**DOI:** 10.18632/oncotarget.24574

**Published:** 2018-02-26

**Authors:** Masahide Kita, Kenji Yokota, Chihiro Kageyama, Susumu Take, Kazuyoshi Goto, Yoshiro Kawahara, Osamu Matsushita, Hiroyuki Okada

**Affiliations:** ^1^ Department of Gastroenterology and Hepatology, Graduate School of Medicine, Dentistry, and Pharmaceutical Science, Okayama University, Okayama, Japan; ^2^ Graduate School of Health Science, Okayama University, Okayama, Japan; ^3^ Department of Bacteriology, Graduate School of Medicine, Dentistry, and Pharmaceutical Science, Okayama University, Okayama, Japan; ^4^ Department of Endoscopy, Okayama University Hospital, Okayama, Japan

**Keywords:** CD14, DEC205, Helicobacter pylori, macrophage, Immunology

## Abstract

*Helicobacter pylori* infections cause gastritis and affect systemic immune responses; however, no direct association between immune cells and stomach bacteria has yet been reported. The present study investigated DEC205-mediated phagocytosis of *H. pylori* and the role of DEC205-positive macrophages in the human gastric mucosa. DEC205 mediated phagocytosis of *H. pylori* was detected immunocytochemically in PMA-stimulated macrophages differentiated from NOMO1 cells. Expression of DEC205 mRNA in peripheral blood mononuclear cells (PBMCs) from *H. pylori*-infected patients was analyzed following stimulation with *H. pylori* cell lysate. We found that anti-DEC205 antibodies inhibited phagocytosis of *H. pylori*. The number of cells double-positive for DEC205 and CD14 in human gastric mucosa was higher in *H. pylori*-infected patients. DEC205-positive macrophages invaded the extracellular space between epithelial cells within gastric pits. In addition, DEC205 mRNA expression was upregulated in human PBMCs stimulated with *H. pylori* lysate. These findings suggest DEC205-expressing macrophages are important for recognition of *H. pylori* in human gastric mucosa, which affects systemic immunity.

## INTRODUCTION

*Helicobacter pylori* is a Gram-negative spiral bacterium that infects the gastric mucosa. It is associated not only with gut diseases such as peptic ulcer, gastric mucosa-associated lymphoid tissue (gastric-MALT) lymphoma, and gastric cancer [1–4], but also with systemic diseases [5, 6]. The mechanism underlying local and systemic acquired immunity in gastric mucosa remains unclear, although gastric-MALT does not occur in healthy mucosa. In a mouse model, antigen-presenting cells (APCs) such as dendritic cells (DCs) within intestinal Peyer’s patches are important for accumulation of CD14-positive cells within gastric mucosa [7], while macrophage-derived cytokine production in the gastric mucosa is strongly upregulated during *H. pylori* infection [8]. These adaptive responses are also characterized by increased lymphocyte and macrophage infiltration of infected gastric tissues [9, 10]. Thus, macrophages and DCs within the gastric mucosa may play an important role in *H. pylori*-induced acquired immunity. However, the mechanisms underlying *H. pylori* recognition and its interaction with the gastric mucosa remain unclear.

DEC205 belongs to the mannose receptor family, a subgroup of the C-type lectin superfamily, and is expressed by macrophages, DCs and B cells, as well as by thymic, pulmonary and intestinal epithelia [11]. In DCs, DEC205 functions as an antigen-uptake receptor that targets its cargo to intracellular compartments to be processed for presentation to T cells [12, 13]. Human DEC205 was initially identified as a 200-kDa glycoprotein (gp200) recognized by the MR6 monoclonal antibody and is expressed by epithelial cells of the thymic cortex, in DCs and, at low levels, in T cells [14]. MR6 was shown to have an anti-proliferative effect on interleukin (IL)-4-dependent T helper 2 immunity [15–17]. DEC205 and gp200 have since been confirmed to be the same molecule based on sequence analysis, and have been designated CD205. However, the distribution of DEC205 within the gastric mucosa has not yet been reported.

In the present study, we investigated the role of DEC205 on macrophages in *H. pylori* infection. The interaction between DEC205 and *H. pylori* was examined using the NOMO-1 human monocyte line, which was stimulated with phorbol 12-myristate 13-acetate (PMA) to induce macrophage differentiation. DEC205-mediated phagocytosis was evaluated using an inhibitory anti-DEC205 antibody. In addition, DEC205-positive macrophages were detected in the gastric mucosa of patients with gastritis, and DEC205 mRNA expression in systemic lymphocytes was evaluated. Our results suggest increasing levels of the endocytic receptor DEC205 in local and systemic immune cells of patients with *H. pylori* infection may mitigate gastric inflammation and enhance systemic immunity.

## RESULTS

### Phagocytosis of *H. pylori* by macrophages via DEC205

**Figure 1:**
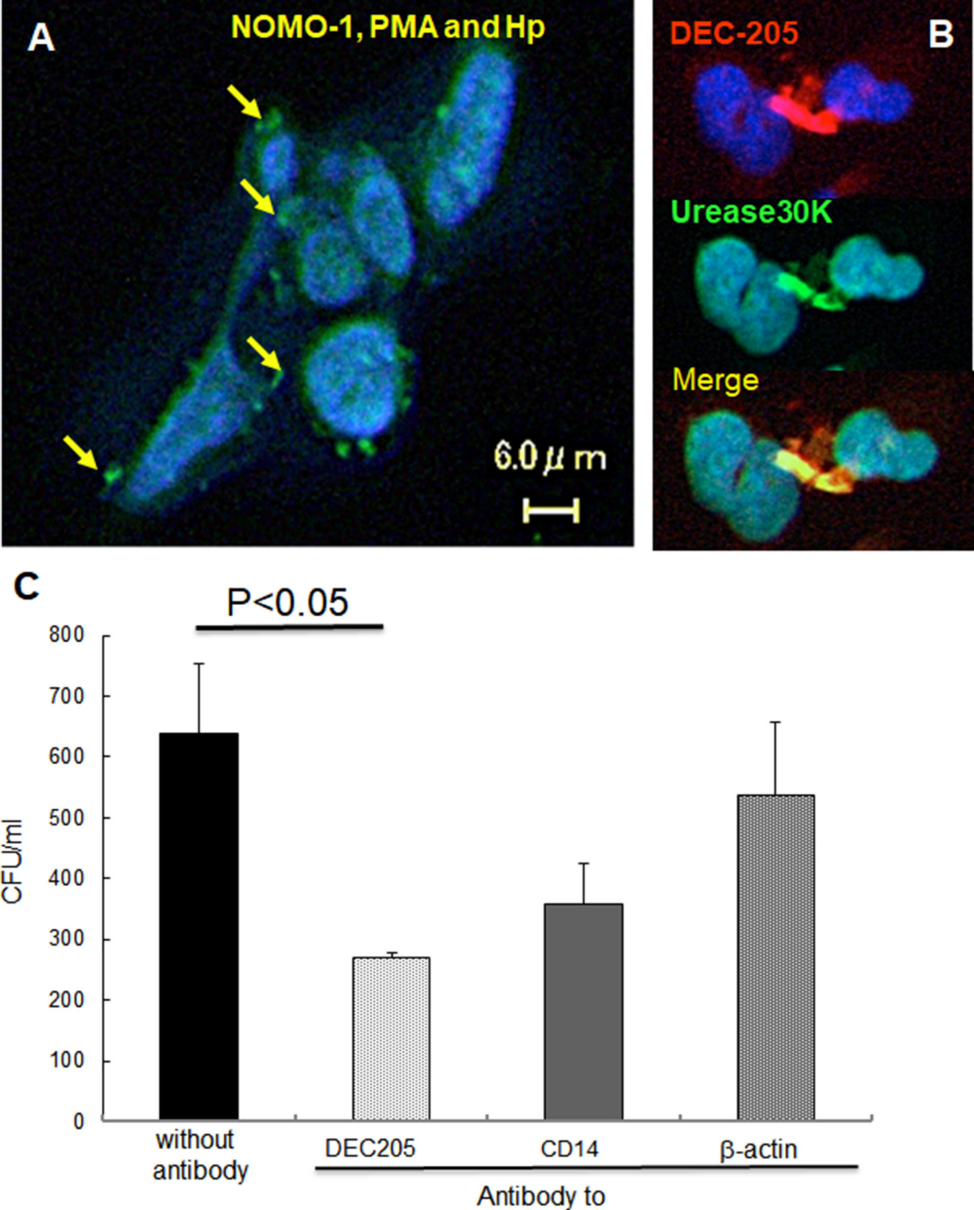
DEC-205′s expression on NOMO-1 cells following *H. pylori* infection and effect on phagocytosis (**A**) Live *H. pylori* were incubated with NOMO-1 cells for 1 hr, and then stained with anti-*H. pylori* urease antibody. Phagocytosed intracellular bacteria were observed. (**B**) Double staining with anti-DEC205 and anti-urease 30 kDa antibodies was performed. Anti-DEC205 mouse monoclonal antibody and anti-urease rabbit antibody were detected with TRITC labeled anti-mouse IgG and FITC labeled anti-rabbit IgG, respectively. (**C**) Inhibition of phagocytosis by pre-treatment with anti-DEC205, anti-CD14, or β-actin antibodies was measured (mean + SE). Anti-DEC205 monoclonal antibody significantly (*p* = 0.03) decreased phagocytosis while CD14 (*p* = 0.09) and β-actin (*p* = 0.28) were not.

We first investigated whether DEC205 plays a role in phagocytosis of *H. pylori*. When *H. pylori* cells were labeled with an antibody against urease (30 kDa), an *H. pylori* surface protein, the bacterial cells were observed within the cytoplasm of NOMO-1 cells (Figure 1A). Double labeling revealed cells that were double-positive for urease and DEC205 (Figure 1B). Phagocytosis was perturbed by treating NOMO-1 cells with anti-DEC205, anti-CD14, or anti-β-actin antibodies prior to *H. pylori* infection (Figure 1C). The number of internalized bacteria was significantly decreased by treatment with anti-DEC205 but not with anti-CD14 or anti-β-actin antibodies.

### DEC205 expression in *H. pylori*-positive human gastric mucosa

**Figure 2:**
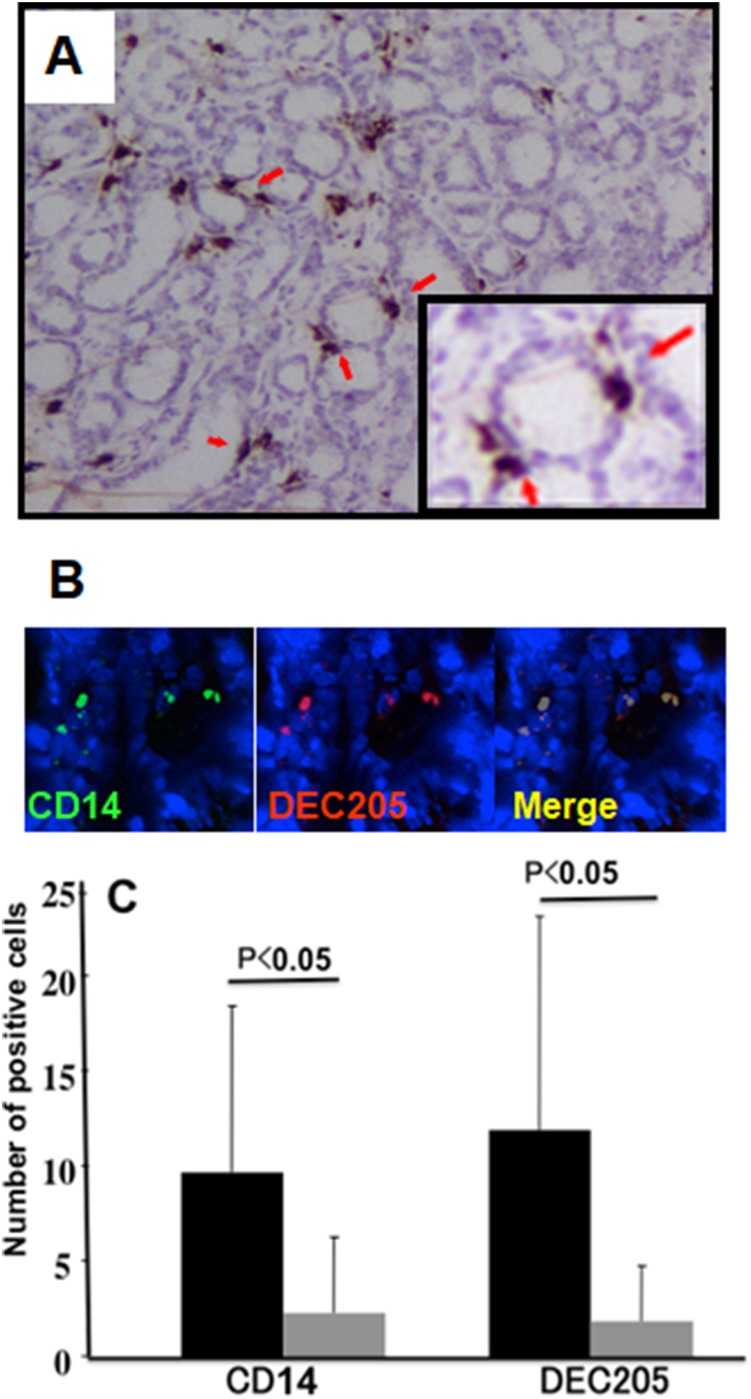
Expression of DEC-205 on macrophages in human gastric mucosa (**A**) Samples were stained with an anti-DEC-205 antibody and observe the location of DEC-205-positive cell in the gastric mucosa. (**B**) Frozen sections of the *H. pylori*-infected gastric mucosa were stained by immunofluorescence double staining. DEC-205 positive cells were stained with mouse monoclonal antibody and detected with a TRITC labeled second antibody. CD14-positive macrophages were detected by anti-CD14 sheep polyclonal antibody by an FITC labeled secondary antibody. More than 90% cells were double positive for CD14 and DEC205. (**C**) The number of positive cells of CD14 and DEC205 in each section (mean positive cell number from 5 fields) was counted before and after eradication. *H. pylori* positive samples are denoted by (black bar: CD14 positive cells; 9.83 ± 8.80, DEC-205 positive cells; 11.88 ± 11.20) and samples obtained after successful eradication are denoted by gray bar: (CD14 positive cells, 2.90 ± 4.63 and DEC-205 positive cells 1.88 ± 2.87). Results were expressed as mean ± SE, and statistical significance was set at *p* < 0.05 (Student’s *t*-test).

We investigated whether DEC205-expressing epithelial cells or macrophages were present within the *H. pylori*-infected gastric mucosa by immunolabeling with an anti-DEC205 antibody. DEC205-positive cells were observed on the surface of gastric pits, between epithelial cells (Figure 2A, red arrows). These DEC205-positive cells also expressed CD14, which confirmed they were macrophages (Figure 2B).

To determine whether DEC205-positive macrophages were induced by *H. pylori* infection, we determined the number of DEC205- and CD14-positive macrophages within the infected mucosa before and after eradication therapy. All CD14-positive cells expressed DEC205, while a few cells that were positive for DEC205 only were deemed to be lymphocytes (B cells) based on their morphology. Eradication therapy markedly reduced the number of DEC205-positive cells (Figure 2C).

### Expression of DEC205 mRNA in human PBMCs

**Figure 3:**
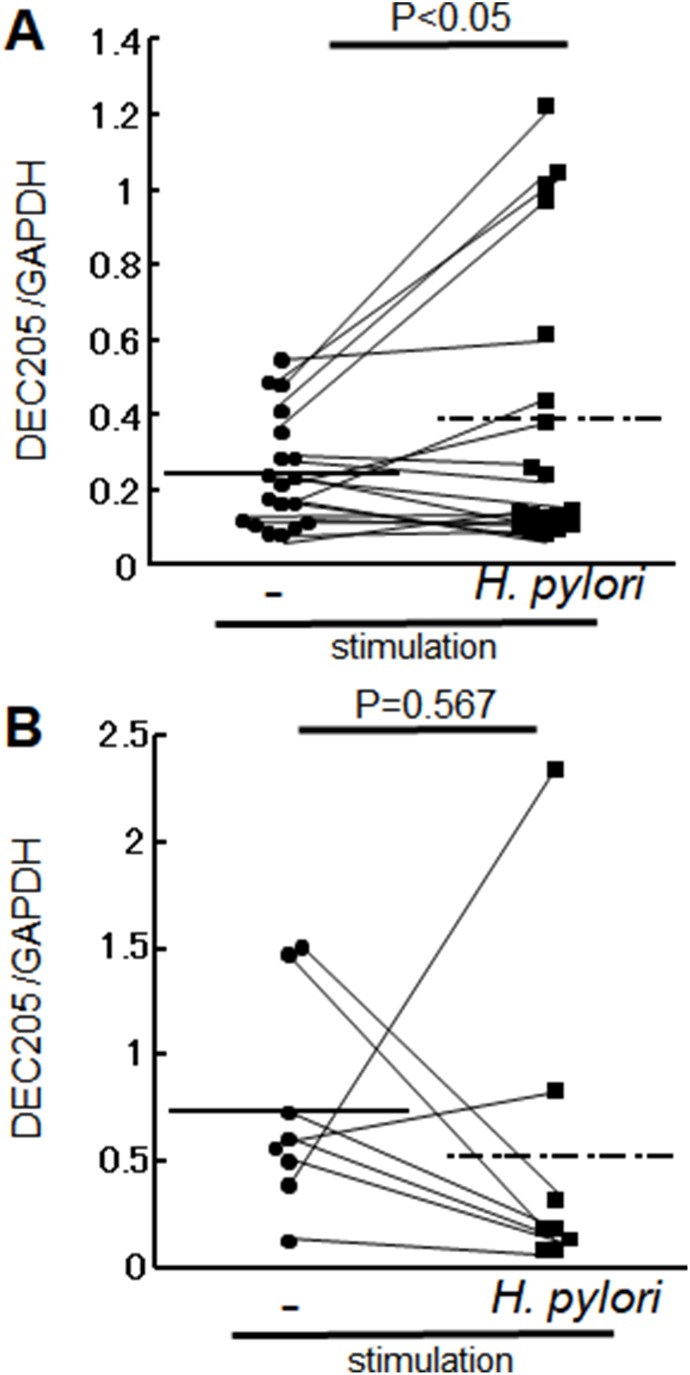
DEC205 mRNA level in PBMCs PBMCs were collected from *H. pylori* infected patients (*n* = 19) and healthy volunteers (*n* = 8). DEC205 mRNA expression was investigated in both groups with and without stimulation with *H. pylori* lysate. DEC205 transcript levels in PBMCs are shown as the ratio between DEC205 and the housekeeping gene GAPDH. (**A**) Transcript levels in patients with gastritis were 0.42 ± 0.14 without stimulation and 0.38 ± 0.38 with *H. pylori* stimulation (*p* < 0.05). (**B**) Transcript levels in healthy volunteers were 0.74 ± 0.49 without stimulation with *H. pylori* stimulation (*p* = 0.567).

We next investigated whether *H. pylori* affects systemic immunity in humans by comparing levels of DEC-205 transcript in circulating PBMCs before and after stimulation with *H. pylori* cell lysate in healthy individuals and *H. pylori*-infected patients with gastritis. Antigen stimulation with *H. pylori* lysate significantly increased levels of DEC205 transcript in the gastritis patients (Figure 3A) but not the healthy individuals (Figure 3B).

## DISCUSSION

It remains unclear how the immune system directly recognizes *H. pylori* infection and reacts to bacteria in the stomach, where MALT is not normally present. Immune responses normally originate within organized intestinal lymphoid tissue (mostly located in the terminal ileum, including Peyer’s patches (PPs) [18]. PPs in the small intestine play critical roles in *H. pylori*-induced gastritis; no gastritis is induced in *H. pylori*-infected mice lacking PPs. Moreover, *H. pylori* converts to the coccoid form in the anaerobic environment of the small intestine and stimulates the host immune system through PPs. [7]. In the present study, we found that DEC205 is expressed in macrophages of the gastric mucosa. DEC205 is internalized from the cell surface by clathrin-mediated endocytosis, and is targeted to late endosome/lysosomes containing major histocompatibility complex (MHC) class II molecules [19]. DEC205 loads antigens on MHC class II molecules for presentation to T lymphocytes [16]. Therefore, a specific immune response acting via DEC205-positive APCs such as DCs (i.e., infiltrating macrophages between epithelial cells) and may be present in the *H. pylori*-infected human gastric mucosa.

Mannose receptor DEC205 is expressed on macrophages and binds to a wide variety of microorganisms, including *Pneumocystis carinii* [20] and *Mycobacterium tuberculosis* [21]. Here, we revealed an association between DEC205 expression in macrophages and human *H. pylori* infection – i.e., DEC205 may participate in phagocytosis of *H. pylori* surface antigens. For example, urease, HSP60, outer membrane protein (oipA), and lipopolysaccharide (LPS) all reportedly affect host cells [18, 19, 22, 23] and may be phagocytosed by macrophages via DEC205.

In the present study, antibodies against DEC205 effectively inhibited phagocytosis of *H. pylori* by NOMO-1 cells. Additionally, Toll-like receptor-mediated signaling reportedly increases DEC205 expression in plasmacytoid DCs [24]. Thus, the innate immune response to several *H. pylori* antigens may induce acquired immunity to *H. pylori* via DEC205. While our study and others [23] suggest *H. pylori* antigens are important for induction of DEC-205 expression, future studies will be need to investigate which antigens are specifically involved.

In sum, we found that *H. pylori* is internalized by macrophages via DEC205, and that the number of DEC205-expressing macrophages is increased in *H. pylori*-positive gastric mucosa. We also found that DEC205 expression by circulating PBMCs from patients with *H. pylori*-induced gastritis is increased following stimulation with *H. pylori* antigens. These results indicate that DEC205 expression is important for the immune response to *H. pylori* infection.

## MATERIALS AND METHODS

### Bacterial strain, antigens, and antibodies

*H. pylori* cells (American Type Culture Collection no. 43504) were cultured on brain heart infusion (BHI) agar supplemented with 7% sterile defibrinated horse blood at 37°C in an incubator containing 10% CO_2_ at 99% humidity. After 4 days of incubation, cells were collected in Roswell Park Memorial Institute (RPMI) 1640 medium (Invitrogen, Carlsbad, CA, USA) without fetal bovine serum (FBS) and resuspended at an OD_600_ of 1.0, corresponding to approximately 1 × 10^8^ CFU/ml. to stimulate human peripheral blood mononuclear cells (PBMCs), *H. pylori* lysate was prepared using ultrasonic sonication of bacteria cultured in BHI broth. The lysate was purified by centrifugation at 20,000 rpm for 20 min, and the supernatant was used as an antigen. An antibody against *H. pylori* urease (30-kDa subunit) was prepared as previously described [25, 26, 27].

### Human tissue and PBMC samples

Patients (*n* = 13) at Nippon Kokan Fukuyama Hospital with *H. pylori*-positive gastritis underwent eradication therapy [28]. Biopsy samples were subsequently obtained after 3 or 6 months via endoscopy from seven of the 13 patients and stored at −80°C until use; these samples were negative for *H. pylori.* PBMCs were collected at Okayama University Hospital from healthy individuals (*n* = 8) and patients with gastritis (*n* = 19). PBMCs were cultured with IL-4 (20 ng/ml; PeproTech, Rocky Hill, NJ, USA) and granulocyte macrophage colony-stimulating factor (GM-CSF) (20 ng/ml; PeproTech) and stimulated with *H. pylori* lysate (5 mg/ml) to induce antigen-specific DCs. Ethical approval to carry out the study was obtained a priori from the Okayama University Ethics Committee (Number 2034).

### Cell culture

The NOMO-1 human monocytic line was obtained from the Japanese Collection of Research Bioresources Cell Bank (Tokyo, Japan) and maintained in RPMI 1640 medium supplemented with 10% FBS, penicillin (5 IU/ml), streptomycin (5 μg/ml), and amphotericin B (2.5 μg/ml) (MP Biomedicals, Santa Ana, CA, USA) at 37°C under an atmosphere of 5% CO2 at 99% humidity. Cells were washed three times with RPMI 1640 without antibiotics before each experiment and used at a final concentration of 2 × 10^6^ cell/ml. To induce macrophage differentiation to DCs, PMA (20 ng/ml) was added to the culture medium 6 h before the experiment.

### Immunocytochemistry

#### Cultured cells

To label engulfed bacteria, NOMO-1 cells (10^6^ cell/ml) were pretreated with PMA for 6 h and then infected with live *H. pylori* (10^7^ colony formation unit; CFU/ml) for 1 h. After washing with phosphate-buffered saline (PBS), cells were fixed with 90% methanol and 10% glycerol at −20°C for 30 min and blocked in 10% fetal calf serum for 1 h at room temperature. Cells were treated with 0.1% Triton X-100 in PBS, followed by incubation for 1 h with anti-DEC205 (BD Biosciences, Franklin Lakes, NJ, USA) and in-house anti-urease (30 kDa) antibodies. Immunoreactivity was detected using tetramethylrhodamine (TRITC)-conjugated anti-mouse IgG and fluorescein isothiocyanate (FITC)-conjugated anti-rabbit IgG, respectively. Cells were imaged using a BZ-9000 Biorevo microscope system (Keyence, Osaka, Japan).

#### Tissue

Frozen sections of gastric mucosa were fixed in acetone and blocked with 10% goat serum for 30 min at room temperature. The blocked sections were incubated first with mouse anti-humanDEC205 and sheep anti-human CD14 antibodies for 1 h at 37°C and then with anti-mouse IgG-TRITC and anti-sheep IgG-FITC for 1 h at room temperature. Nuclei were counterstained with 4’,6-diamidino-2-phenylindole. Tissue sections were imaged using a BZ-9000 Biorevo microscope system.

### Detection of the intracellular bacterial count

A phagocytosis assay was carried out as previously described [29], with modifications. NOMO-1 cells (1 × 10^6^ cell/ml) were induced undergo differentiation for 12 h using PMA. Differentiated cells were infected with 5 × 10^7^ live *H. pylori* /ml for 30 min, followed by incubation for 5 min with RPMI 1640 medium containing penicillin (100 U/ml) and streptomycin (100 U/ml) to kill extracellular bacteria. The cells were then washed four times with RPMI 1640 medium without antibiotics. To harvest the intracellular bacteria, cells were osmotically lysed in distilled water. A 10-μl volume of the cell lysate was inoculated on BHI blood agar under microaerophilic conditions. Colonies were counted after culturing for 7 days. For inhibition using antibodies, NOMO1 cells were pretreated for 30 min with 1 μg/ml anti-DEC205, anti-CD14 (R&D Systems, Minneapolis, MN, USA), or anti-β-actin (Novus Biologicals, Littleton, CO, USA) antibodies.

### Real-time reverse-transcriptase (RT-)PCR

Total RNA was extracted from PBMCs (approximately 3 × 10^5^ cells) using a RNeasy Mini kit (Qiagen, Valencia, CA, USA). RNA concentration and purity were determined by measuring the absorbance at 260 nm (A260) and the A260/A280 ratio using a DU-7000 spectrophotometer (Beckman Coulter, Brea, CA, USA). For cDNA synthesis, reverse transcription was carried out using a SuperScript III First-Strand Synthesis system (Invitrogen). To ensure the RNA samples were free of DNA contamination, the assay was performed in duplicate, omitting reverse transcriptase in one set of samples. Real-time PCR was carried out in a Light Cycler Quick System 350S (Roche Diagnostics, Indianapolis, IN, USA). cDNA (2 μl) was amplified in 20-μl of reaction mixture containing 10 μl of SYBR Premix Ex Taq (Takara Bio, Otsu, Japan), 2 μl of primer for DEC205 (5′-CAAATTCCAAAAGGCCGTACTC-3′ and 5′-CACCACTTCTGTCCATCACCA-3′) or glyceraldehyde 3-phosphate dehydrogenase (GAPDH) (5′-CAACGGATTTGGTCGTATTGG-3′ and 5′-CTGGAAGATGGTGATGGGATTT-3′) as a control, and 7.2 μl of distilled water. The reaction conditions were as follows: 95°C for 30 s; 35 cycles of 95°C for 5 s, 55.5°C for 10 s, and 72°C for 15 s; 65°C for 15 s; and 40°C for 30 s. Data were analyzed using Light Cycle software v.3.5 (Roche Diagnostics), and the ratio of DEC205/GAPDH mRNA was calculated. Differences between samples were evaluated using Student’s *t* test with significance set at *P* < 0.05.

### Statistics

Phagocytosis assays were analyzed using a post-hoc test, and DEC205 mRNA expression levels were analyzed using paired *t*-test. Values of *P* < 0.05 was regarded as significant.

## Abbreviations

PBMC: Peripheral blood mononuclear cell
APC: antigen-presenting cell
DC: dendritic cell
PBS: phosphate-buffered saline
IL: interleukin
CFU: colony formation unit
